# Development of clinical prediction rule for diagnosis of autistic spectrum disorder in children

**DOI:** 10.1108/MIJ-01-2020-0001

**Published:** 2020-03-20

**Authors:** Tiraya Lerthattasilp, Chamnan Tanprasertkul, Issarapa Chunsuwan

**Affiliations:** Faculty of Medicine, Thammasat University – Rangsit Campus, Khlong Luang, Thailand

**Keywords:** Autistic spectrum disorder, Autism, Autistic, Prediction, Diagnosis

## Abstract

**Purpose:**

This study aims to develop a clinical prediction rule for the diagnosis of autistic spectrum disorder (ASD) in children.

**Design/methodology/approach:**

This population-based study was carried out in children aged 2 to 5 years who were suspected of having ASD. Data regarding demographics, risk factors, histories taken from caregivers and clinical observation of ASD symptoms were recorded before specialists assessed patients using standardized diagnostic tools. The predictors were analyzed by multivariate logistic regression analysis and developed into a predictive model.

**Findings:**

An ASD diagnosis was rendered in 74.8 per cent of 139 participants. The clinical prediction rule consisted of five predictors, namely, delayed speech for their age, history of rarely making eye contact or looking at faces, history of not showing off toys or favorite things, not following clinician’s eye direction and low frequency of social interaction with the clinician or the caregiver. At four or more predictors, sensitivity was 100 per cent for predicting a diagnosis of ASD, with a positive likelihood ratio of 16.62.

**Originality/value:**

This practical clinical prediction rule would help general practitioners to initially diagnose ASD in routine clinical practice.

## Introduction

Autistic spectrum disorder (ASD) is a neurodevelopmental disorder with a prevalence of 1:68 in children (Christensen, 2016; American Psychiatric Association, 2013). Early diagnosis with early intervention yield ameliorated long-term outcomes (Filipek *et al.*, 2000; Granpeesheh *et al.*, 2009; Landa, 2008). Because ASD is a disorder with a multitude of signs and symptoms, the diagnosis process requires massive history taking from the caregiver together with time-consuming clinical observation by experienced clinicians (Wing, 1988; Falkmer *et al.*, 2013). Doctors working in countries with inadequate specialists and resources are challenged by the ASD diagnosis. These general practitioners, limited by time and experience, may underdiagnose ASD, resulting in delayed treatment. Having clear and concise predictors to facilitate the initial diagnosis of ASD in busy clinical practice would benefit both doctors and patients (Zwaigenbaum *et al.*, 2015). This study aimed to develop a prediction rule for the diagnosis of ASD in children from baseline characteristic profiles, risk factors, history and clinical observation.

## Materials and methods

We conducted a population-based study from January to December 2018 in consecutive children aged 2-5 years suspected of ASD who visited Thammasat University Hospital. Eligibility was based upon the patients having any one of the following chief complaints: delayed speech (no discrete words by 18 months or no phrases by 24 months or no complete sentences speech by 36 months); social or play problems, e.g. preferred to be left alone; repetitive behaviors or restricted interests; behavioral or emotional regulation problems; or doctors/parents concerned that the child may have had ASD. Patients were excluded if they had any of the followings: severe chronic medical illness or physical disability, congenital anomalies/syndromes or hearing problems, had already been diagnosed with ASD, the main caregiver did not attend with the child and the caregiver was not able to communicate in Thai.

### Assessment and data collection

The potential predictor variables included demographic data and risk factors, i.e. gender, age, chief complaint, level of communication, birthweight, maternal and paternal age, family history of autism or developmental delay, caregiver level of education, history of child’s ASD symptoms and symptoms from clinical observation (Appendix). All variables were selected based upon a review of the existing literature (Devlin and Scherer, 2012; Ozonoff *et al.*, 2011; Gardener *et al.*, 2009; Hultman *et al.*, 2011; Ozonoff *et al.*, 2009; McCoy *et al.*, 2009; Clifford *et al.*, 2013; Allison *et al.*, 2012; Srisinghasongkram *et al.*, 2016; Pornnoppadol *et al.*, 2002; Panyayong, 2011; Krivichian, 2014; Maenner *et al.*, 2013; Tsheringla *et al.*, 2014; Dow *et al.*, 2017; Ozonoff *et al.*, 2008; Watt *et al.*, 2008). While the caregiver filled out form on the demographic specifics, risk factors and history of child’s ASD symptoms, a general practitioner observed patients’ symptoms according to a prepared checklist. Both steps took less than 20 minutes per patient. All patients, then, were independently assessed by trained research assistants using ASD standardized diagnostic tools (Huerta and Lord, 2012). The Developmental, Dimensional and Diagnostic Interview short form and Autism Diagnostic Observation Schedule (Santosh *et al.*, 2009; Chuthapisith *et al.*, 2012; Lord *et al.*, 2000). ASD diagnosis was made, in accordance with The *Diagnostic and Statistical Manual of Mental Disorders*, Fifth Edition (DSM-5), by a child psychiatrist or developmental and behavioral pediatrician using clinical assessment and information from both tools. Other diagnosis and comorbidities were given following the DSM-5 criteria.

### Data analysis

ASD and non-ASD groups were compared for evidence of differences (*p-*value) in clinical characteristics with *t*-test or exact probability test as appropriate. Prediction by each characteristic was calculated using univariable logistic regression and presented as an area under the receiver operating characteristic (AuROC) curve and its 95 per cent confidence interval (95% CI). Clinical predictors with a high AuROC curve and *p* value <0.01 were selected and processed with multivariable logistic regression with backward stepwise selection (*p* < 0.1) to aid the selection of the best variables. The discriminative performance of the model was calculated by an AuROC curve. The regression coefficient of each clinical predictor was divided by the smallest coefficient of the model and transform into an item risk score. Scores for each clinical predictor were added up to obtain a total risk score. Score prediction of ASD diagnosis was done by using a total score as the only summary predictor in the logistic model. Discrimination of the score was presented with an AuROC curve. Calibration of the prediction was analyzed with Hosmer–Lemeshow statistics. Scores predicting risk and observed risk were compared and presented in a graph. Internal validation of the score was done by logistic regression with the bootstrap method. Risk scores were categorized into risk levels. The predictive ability of each risk score level was calculated and presented as a likelihood ratio of positive, 95% CI and its significance level. This research was approved by the research ethics committees of the Faculty of Medicine, Thammasat University.

## Results

One hundred and thirty-nine patients were enrolled (Table I). All patients had a complete assessment of ASD, and 104 (74.8 per cent) were diagnosed with ASD. In non-ASD group, diagnoses were language disorder (7.9 per cent), attention-deficit hyperactivity disorder (7.9 per cent), typical development (5.0 per cent), global developmental delay (2.9 per cent) and childhood-onset fluency disorder (stuttering) (1.5 per cent).

Eighty-five predictors from the patient profile, history taking and clinical observation were assessed. The association between all predictor variables and diagnosis of ASD determined using univariate analyses and the prediction ability measured by using AuROC were shown in the Appendix. Predictors that had *p* <* 0.01 from univariate analyses were the level of communication, 11 symptoms from history taking and 16 symptoms observed by the clinician (Table II). These 28 variables were processed with multivariable logistic regression with backward stepwise selection (*p* < 0.1).

### Prediction model

The best multivariable clinical predictors for the diagnosis of ASD from the multiple logistic regression were level of communication, history of rarely making eye contact or looking at faces, history of not showing off toys or favorite things, did not follow the clinician’s eye direction when called and signaled with eyes to look at things far away and had low frequency of social interaction with the clinician or the caregiver in the room. These five clinical predictors were each categorized into two levels. An item score of 1 was assigned to each predictor (Table III).

**Figure 1:**
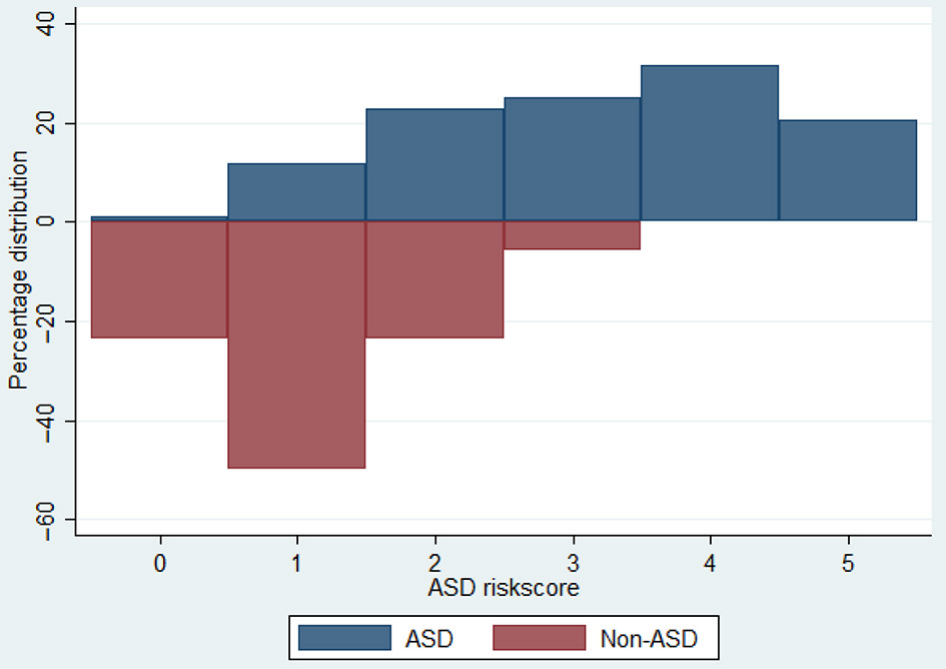
Percentage distribution of clinical risk score of ASD (*n* = 104) and non-ASD (*n* = 35)

A summary risk score was obtained by adding up the item scores. The discriminative ability of the derived risk score, which ranged from 0 to 5, could directly be observed by the different percentage distribution between ASD and non-ASD groups (Figure 1).

**Figure 2:**
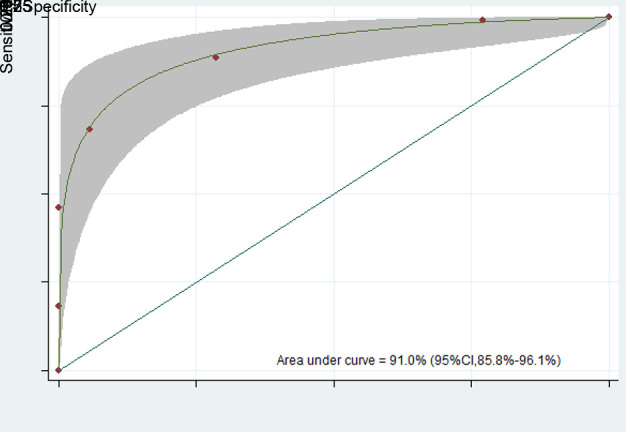
Area under receiver operating characteristic curve of clinical risk score and 95% confidence interval (CI) on prediction of ASD diagnosis

The risk score predicted a diagnosis of ASD with an AuROC curve of 91.0 per cent (95% CI, 85.8-96.1) (Figure 2) and with the *p-*value for the Hosmer–Lemeshow goodness-of-fit test of 0.67. Internal validation by the bootstrapping method (1,000 replications) reduced the AuROC curve to 83.26 per cent (95% CI, 76.0-90.5).

**Figure 3:**
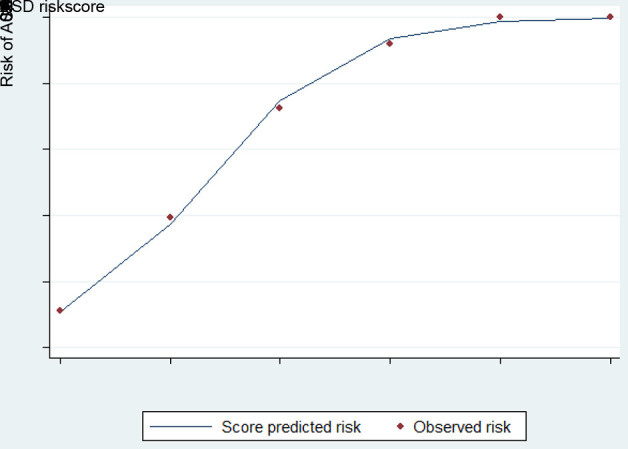
Observed risk (circle) vs score predicted risk (solid line) of ASD diagnosis

When translating into absolute risks, the score predicted the risk of diagnosis of ASD increased when the risk score moved upward, with close calibration to the actual or observed risks (Figure 3).

The risk scores were categorized into three risk groups, low (0) when the slope of the risk curve was lowest, moderate (1-3), and high (4-5) to facilitate clinical interpretation. The positive likelihood ratio for the diagnosis of ASD was 0.04 in the low risk, 0.45 (95% CI, 0.34-0.59) in the moderate and 16.62 (95% CI, 2.38, 116.05) in the high categories (Table IV).

## Discussion

This clinical decision rule has been developed to help general practitioners for predicting the diagnosis of ASD in children aged 2-5 years old.

Research in the past from the UK found that parents of children with ASD brought them to hospital from age 2 ± 1.92 years, but the average age of diagnosis was 5.7 years. In the first visit, usually with a general practitioner, less than 10 per cent of patients received diagnosis, and 26-30 per cent were told “no problem/no worry”. The other 50 per cent were referred to specialists (Howlin and Asgharian, 1999). This older study may convey the situation in Thailand and other developing countries today. Furthermore, in these countries where specialists are less than adequate, the referring process may take years. Caregivers who are not confident in the diagnosis may be lost to follow-ups, and the early intervention will be delayed. This clinical decision rule would allow the general practitioners to make the initial diagnosis of ASD based upon the clear and evidenced rule. Having more confidence regarding the initial diagnosis, they are able to provide disease-specific initial recommendations and management for caregivers and families.

Recently, several screening questionnaires for autism have been developed (Allison *et al.*, 2012; Srisinghasongkram *et al.*, 2016; Pornnoppadol *et al.*, 2002; Panyayong, 2011; Krivichian, 2014). This decision rule would facilitate the initial diagnosis in patients with positive result from the screening process. This risk score is highly accurate in the predicted diagnosis of ASD (the AuROC being 91.0 per cent). We chose the cutoff score of 4 to classify patients into a very high-risk group. We chose a high cutoff score because we want this decision rule to be highly specific so the doctors would be confident in the initial diagnosis.

To apply this rule in practice, patients with four or more of these predictors, namely:

- delayed speech for their age;
- history of avoiding eye contact/meeting others’ gaze;
- history of a pattern of not showing objects to others;
- poor response when the clinician attempts to draw attention to something in a distance; and
- low frequency in reciprocal social interaction with the clinician or the caregiver in the room are at substantial risk of having ASD (positive likelihood ratio = 16.62).

A doctor can discuss the ASD diagnosis and give psychoeducation to the family. Also, initial management can be done promptly, i.e. referral to a speech therapist, occupational therapist or developmental stimulation program. Patients with one to three predictors may or may not have ASD and should be referred to specialists. Patients with no predictors are at low risk of having ASD. They can be managed as per other diagnoses or observed.

The strength of this study is that it was a population-based study conducted in routine clinical practice with limited observation time. Patients and doctors would represent target groups that results were intended to be used. The results also showed which ASD symptoms can be observed in the time-limited outpatient situation. The diagnosis process was based on the reference standard for the ASD diagnosis. As all variables were collected before the specialist assessed the patients, the bias of information would be reduced. Furthermore, as the format of the rule includes a simple list of history taking and clinical observations, it would make this rule clinically sensible for the busy general practitioner to apply it in routine practice.

However, the number of patients in this study was small, and the derived score is likely to be space domain specific. Also, as all data were collected in Thai, cultural and language effects should be considered. Clinical predictors in our setting may not be directly applicable to other settings. Model adjustment, either selection of different clinical predictors and/or different scoring weights, should always be considered for application to a new setting. Also, it is necessary for the model to have an external validation to provide sufficient evidence about its performance.

## Conclusion

This simple and practical clinical decision rule may help non-specialists to make the initial diagnosis of ASD in children. Caregivers of the very high-risk patients may be informed about the disease and its caring process that will improve the quality of care.

## Figures and Tables

**Table I:** Characteristic of the patients

**Characteristics**	No. of patients (%)
**Men**	119 (85.6)
**Mean age in months (SD)**	44 (9.6)
**Age range in months**	25-60
**Chief complaints**	
**Delayed speech** ** Social or play problems** **Repetitive behaviors or restricted interests** **Behavioral or emotional regulation problems** ** Doctors/parents suspected ASD**	84 (60.4) 12 (8.6) 5 (3.6) 33 (23.7) 5 (3.6)
**Level of communication**	
**No meaningful word**	7 (5.0)
**Discrete words**	56 (40.3)
**Phrase**	35 (25.2)
**Complete sentence**	41 (29.5)
**Mean caregiver education in year (SD)**	12 (4.6)
**Year of education range**	0-19

**Table II:** Univariate correlation values of variables and area under receiver operating curve (AuROC) and 95% confidence interval (CI) from patients’ profiles, caregiver report and clinical observation

**Predictors**	ASD (%)	Non-ASD (%)	*p*-value	AuROC
***Patients’ characteristic***				
**Level of communication**			<0.01	0.77 (0.69-0.84)
**No meaningful word**	5 (4.8)	2 (5.7)		
**Discrete words**	54 (51.9)	2 (5.7)		
**Phrase**	26 (25.0)	9 (25.7)		
**Complete sentence**	19 (18.2)	22 (62.8)		
***Caregiver report***				
**Can do role-play, such as feeding dolls, acting as a goods vendor or other roles**	60 (57.69)	30 (85.71)	<0.01	0.64 (0.55-0.72)
**Nods or shakes his/her head to let you know that (s)he wants or does not want something**	71 (68.27)	32 (91.43)	<0.01	0.62 (0.53-0.70)
**Takes your hand to get what (s)he wants without looking at your face**	41 (39.42)	25 (71.43)	<0.01	0.66 (0.58-0.74)
**Knows to comfort other children when they are upset or injured**	34 (32.69)	21 (60.00)	<0.01	0.64 (0.55-0.71)
**Rarely makes eye contact or looks at faces, and usually looks another way when talked to**	62 (59.62)	10 (28.57)	<0.01	0.66 (0.57-0.73)
**Does not brag or persuade parents to be interested in what (s)he is doing**	36 (34.62)	3 (8.57)	<0.01	0.63 (0.55-0.71)
**Speaks a language of his/her own**	72 (69.23)	14 (40.00)	<0.01	0.65 (0.56-0.73)
**Shows off toys or favorite things**	60 (57.69)	32 (91.43)	<0.01	0.67 (0.58-0.75)
**Turns to look at you upon you calling his/her name**	81 (77.88)	34 (97.14)	<0.01	0.60 (0.51-0.68)
**Shows off or shows any items to you**	69 (66.35)	32 (91.43)	<0.01	0.63 (0.54-0.71)
**Looks at things you are looking at**	70 (67.31)	32 (91.43)	<0.01	0.62 (0.53-0.70)
***Clinical observation***				
**The child’s eye contact is abnormal**	69 (66.35)	14 (40.00)	<0.01	0.63 (0.55-0.71)
**Gestures and words the child uses to approach you look weird**	78 (75.00)	16 (45.71)	<0.01	0.65 (0.56-0.73)
**When the child’s name is called (without touching), (s)he turns to look at you**	44 (42.31)	28 (80.00)	<0.01	0.69 (0.61-0.77)
**When you call to the child and signal with your eyes for him/her to look at things far away (without touching), (s)he looks in your eyes’ direction to those things**	43 (40.38)	30 (85.71)	<0.01	0.73 (0.65-0.80)
**If you hold a toy the child wants in your hand, (s)he speaks or make gestures along with eye contact to ask for it**	39 (37.50)	25 (71.43)	<0.01	0.67 (0.58-0.75)
**Requesting eye contact, speech and gestures are simultaneous (natural)**	27 (25.96)	19 (54.29)	<0.01	0.64 (0.56-0.72)
**Upon getting toys, the child shows off/shows it to you or guardian**	28 (26.92)	24 (68.57)	<0.01	0.71 (0.62-0.78)
**While playing with toys, the child tries to get attention so that you or guardian become(s) interested in what (s)he is interested in (for mutual interest)**	28 (26.92)	20 (57.14)	<0.01	0.65 (0.57-0.70)
**The child often interacts with you or guardian in examination room, such as makes eye contact, smiles at you or the guardian, initiates conversations or asks questions**	24 (23.08)	27 (77.14)	<0.01	0.77 (0.69-0.83)
**Overall, you can build a natural relationship with the child**	27 (25.96)	21 (60.00)	<0.01	0.67 (0.58-0.75)
**The child has language development for age (can say short phrases by two years of age, can say short sentences by three, can say several consecutive sentences by four)**	16 (15.38)	16 (45.71)	<0.01	0.63 (0.54-0.71)
**The child has natural speaking/tone of voice (that does not sound weird)**	22 (21.15)	16 (45.71)	< 0.0.01	0.62 (0.54.0.71)
**The child uses gestures in communication (such as makes gestures in story-telling, shakes his/her head, nods or waives his/her hand in rejection)**	37 (35.58)	24 (68.57)	<0.01	0.67 (0.58-0.75)
**If you hold one toy in each hand and ask the child which toy (s)he wants, (s)he can point index finger to the toy (s)he wants on his/her own without having to be told**	38 (36.54)	26 (74.29)	<0.01	0.69 (0.61-0.77)
**The child makes noises or strange speech (such as alien language, suddenly speaking out sounds from TV)**	65 (62.50)	10 (28.57)	<0.01	0.67 (0.58-0.75)
**The child is more interested in certain objects in examination room than people**	71 (68.27)	12 (34.29)	<0.01	0.67 (0.58-0.75)

**Table III:** Clinical predictors, odds ratio (OR), 95% confidence interval (CI), logistic regression beta coefficient (*β*) and assigned item scores

**Predictors**	OR	95% CI	*p*-value	*Β*	Score
**Delayed speech for their age**	4.83	1.65-14.15	0.004	1.58	1
**History of rarely making eye contact or looks at faces, and usually looks another way when talked to**	4.81	1.58-14.65	0.006	1.57	1
**History of not showing off toys or favorite things**	5.69	1.18-27.36	0.030	1.74	1
**Did not follow clinician’s eye direction when called and signaled with eyes to look at things far away**	3.22	0.89-11.62	0.075	1.17	1
**Had low frequency of social interaction with clinician or caregiver in the room**	6.74	2.25-20.22	0.001	1.91	1

**Table IV:** Distribution of ASD vs non-ASD into low, moderate to high and very high probability categories, likelihood ratio of positive (LHR+) and 95% confidence interval (CI)

**Probability** **categories**	Score	Case (*n* =104) *n* (%)	Control (*n* = 35) *n* (%)	LHR+	95% CI	*p*-value
**Low risk**	0	1 (11.11)	8 (88.89)	0.04	0.01-0.33	<0.01
**Moderate risk**	1-3	55 (67.07)	27 (32.93)	0.45	0.34-0.59	<0.01
**High risk**	4-5	48 (100.0)	0 (0.0)	16.62	2.38-116.05	<0.01
**Mean ± SD**		1.1 ± 0.8	3.2 ± 1.3			<0.01

**Table AI:** Univariate correlation values of variables and area under receiver operating curve (AuROC) and 95% confidence interval (CI) from patients’ profiles

**Predictors**	ASD	Non-ASD	*p*-value	AuROC (95% CI)
**Male *n* (%)**	88 (84.6)	31 (88.6)	0.78	0.48 (0.40-0.57)
**Age (months)**	43 (0.9)	48 (1.7)	0.01	0.35 (0.27-0.44)
**Birthweight (g)**	3016.2 (61.5)	3038.0 (83.9)	0.85	0.50 (0.42-0.59)
**Level of communication *n* (%)** **No meaningful words** **Discrete words** **Phrases** **Complete sentences**	5 (4.8) 54 (51.9) 26 (25.0) 19 (18.2)	2 (5.7) 2 (5.7) 9 (25.7) 22 (62.8)	<0.01	0.77 (0.69-0.84)
**Family history of ASD *n* (%)**	38 (26.5)	12 (34.3)	0.84	0.51 (0.42-0.60)
**Paternal age (years)**	33.8 (0.9)	33.7 (1.4)	0.94	0.54 (0.45-0.62)
**Maternal age (years)**	30.9 (0.7)	30.8 (1.1)	0.94	0.50 (0.41-0.58)
**Caregiver’s level of education (years)**	12.3 (4.6)	11.9 (4.7)	0.66	0.53 (0.42-0.64)

**Table AII:** Univariate correlation values of variables and area under receiver operating curve (AuROC) with 95% confidence interval (CI) from caregiver reports

**No.**	Caregiver report	ASD *N* (%)	Non-ASD *N* (%)	*p*-value	AuROC (95% CI)
**Deficits in social communication and social interaction**					
***Interaction with parents***					
**1**	**Smiles at parents upon seeing them from a distance**	92 (88.46)	32 (91.43)	0.76	0.52 (0.43-0.60)
**2**	**Smiles upon seeing you or in response to your smiles**	98 (94.23)	32 (91.43)	0.69	0.49 (0.40-0.58)
**3**	**Likes to be hold, hugged or kissed by parents**	95 (91.35)	32 (91.43)	1.00	0.50 (0.42-0.59)
**4**	**Likes to show affections to parents by hugging, kissing or embracing**	93 (89.42)	32 (91.43)	1.00	0.51 (0.42-0.60)
**5**	**Rushes to you to get help or to ask for comfort in times of injuries or accidents**	99 (95.19)	31 (88.57)	0.23	0.47 (0.38-0.55)
**6**	**Seems not troubled or paying attention to having or not having your company**	40 (38.46)	8 (22.86)	0.10	0.58 (0.49-0.66)
**7**	**Does not understand other people’s thoughts, facial expressions or emotions, such as does not realize when parents scold him/her**	21 (20.19)	4 (11.43)	0.31	0.54 (0.46-0.63)
**8**	**Turns to look at you upon you calling his/her name**	81 (77.88)	34 (97.14)	<0.01	0.60 (0.51-0.68)
**9**	**Acts as if not listening when you speak to him/her**	73 (70.19)	18 (51.43)	0.06	0.59 (0.51-0.68)
**10**	**When pointed to things, the child is interested and looks in corresponding directions**	85 (81.73)	33 (94.29)	0.10	0.56 (0.48-0.65)
**11**	**Looks at things you are looking at**	70 (67.31)	32 (91.43)	<0.01	0.62 (0.53-0.70)
**12**	**Tries to make you interested in what (s)he is doing by calling to you or handing it to you**	82 (78.85)	32 (91.43)	0.13	0.56 (0.47-0.65)
**13**	**Does not brag or persuade parents to be interested in what (s)he is doing**	36 (34.62)	3 (8.57)	<0.01	0.63 (0.55-0.71)
**14**	**Shows off toys or favorite things**	60 (57.69)	32 (91.43)	<0.01	0.67 (0.58-0.75)
**15**	**Shows off or shows any items to you**	69 (66.35)	32 (91.43)	<0.01	0.63 (0.54-0.71)
***Interaction with other children***					
**16**	**Interested and wants to play with other children at school or playground**	74 (71.15)	30 (85.71)	0.12	0.57 (0.49-0.66)
**17**	**Likes to play alone, to isolate himself/herself and is not interested in other children**	51 (49.04)	8 (22.86)	0.01	0.63 (0.55-0.71)
**18**	**Knows to share snacks or toys with other children**	74 (71.15)	27 (77.14)	0.53	0.53 (0.45-0.62)
**19**	**Responds appropriately, such as looks at faces or in the eyes, smiles, or hands over toys, when other children approach**	68 (65.38)	26 (74.29)	0.41	0.55 (0.46-0.063)
**20**	**Knows to comfort other children when they are upset or injured**	34 (32.69)	21 (60.00)	<0.01	0.64 (0.55-0.71)
***Non-verbal communication***					
**21**	**Often has glazed eyes or unfocused stares**	32 (30.77)	6 (17.14)	0.13	0.57 (0.48-0.65)
**22**	**Rarely makes eye contact or looks at faces, and usually looks another way when talked to**	62 (59.62)	10 (28.57)	<0.01	0.66 (0.57-0.73)
**23**	**Stares with corners of eyes**	35 (33.65)	5 (14.29)	0.03	0.60 (0.51-0.68)
**24**	**Points index finger to communicate interests**	82 (78.85)	32 (91.43)	0.13	0.56 (0.47-0.65)
**25**	**Takes your hand to get what (s)he wants without looking at your face**	63 (60.58)	10 (28.57)	<0.01	0.66 (0.58-0.74)
**26**	**Nods or shakes his/her head to let you know that (s)he wants or does not want something**	71 (68.27)	32 (91.43)	<0.01	0.62 (0.53-0.70)
**27**	**Straight-faced child, rarely showing emotions**	21 (20.19)	5 (14.29)	0.62	0.53 (0.45-0.62)
***Language, play and imitation***					
**28**	**Was able to speak but no longer speaks**	25 (24.04)	3 (8.57)	0.05	0.58 (0.49-0.66)
**29**	**Delayed speech, meaning not yet able to do any of the followings** ** Does not say meaningful single words, such as mom or eat, at age of 18 months** ** Does not say word groups with at least two words together, such as have meal**	64 (61.54)	12 (34.29)	0.60	0.64 (0.55-0.71)
**30**	**You used to wonder whether (s)he could be deaf**	24 (23.08)	4 (11.43)	0.15	0.56 (0.48-0.65)
**31**	**You used to feel that speech is delayed or to worry why your child does not start to speak**	89 (85.58)	22 (62.86)	<0.01	0.61 (0.53-0.69)
**32**	**Cannot yet communicate what (s)he wants by speaking or pointing**	34 (32.69)	5 (14.29)	0.05	0.59 (0.50-0.67)
**33**	**Understands what others say**	74 (71.15)	31 (88.57)	0.04	0.59 (0.50-0.67)
**34**	**Does not know how to play with toys; taps, smells, throws or tosses them**	36 (34.62)	7 (20.00)	0.14	0.57 (0.49-0.66)
**35**	**Can do role-play, such as feeding dolls, acting as a goods vendor or other roles**	60 (57.69)	30 (85.71)	<0.01	0.64 (0.55-0.72)
**36**	**Can make gestures imitating adults, such as wearing makeups, combing hair, shaving or getting ready to go to work**	81 (77.88)	29 (82.86)	0.64	0.53 (0.44-0.61)
**37**	**Imitates your actions, such as sticks out tongue when you do so at him/her**	74 (71.15)	29 (82.86)	0.19	0.56 (0.48-0.65)
**Restricted, repetitive patterns of behavior, interests or activities**					
***Stereotyped or repetitive motor movements, use of objects or speech.***					
**38**	**Make repeated gestures (such as flick of the hand, tiptoeing, body rotation)**	46 (44.23)	9 (25.71)	0.07	0.59 (0.50-0.67)
**39**	**Likes doing or saying something repeatedly**	65 (62.50)	16 (45.71)	0.11	0.58 (0.50-0.67)
**40**	**Likes to arrange toys in rows and will get very angry if someone re-arranges them**	54 (51.92)	16 (45.71)	0.56	0.53 (0.45-0.62)
**41**	**Speaks a language of his/her own**	72 (69.23)	14 (40.00)	<0.01	0.65 (0.56-0.73)
**42**	**Says words (s)he hears or words on TV; repeats the last word**	59 (56.73)	14 (40.00)	0.12	0.58 (0.50-0.67)
**43**	**Often repeats what you just said**	47 (45.19)	13 (37.14)	0.44	0.54 (0.45-0.62)
***Insistence on sameness, inflexible adherence to routines or ritualized patterns of verbal or nonverbal behavior***					
**44**	**Hard to adapt to new things, such as refuses to try new dishes, cries when going to new places**	38 (36.54)	10 (28.57)	0.42	0.54 (0.45-0.62)
**45**	**Is hard to change what (s)he is used to doing; has own patterns**	31 (29.81)	12 (34.29)	0.67	0.48 (0.40-0.57)
**46**	**Seems like a more “organized” child than his/her peers**	19 (18.27)	5 (14.29)	0.80	0.52 (0.43-0.60)
***Highly restricted, fixated interests that are abnormal in intensity or focus***					
**47**	**Interested in few toys or matters**	52 (50.00)	13 (37.14)	0.24	0.56 (0.48-0.65)
**48**	**Is obsessed with something or always holds something, such as drinking straw or rope**	32 (30.77)	7 (20.00)	0.28	0.55 (0.47-0.64)
**49**	**Interested in playing a particular part of objects, such as car wheel**	44 (42.31)	8 (22.86)	0.05	0.60 (0.51-0.68)
**Hyper- or hyporeactivity to sensory input or unusual interest in sensory aspects of the environment**					
**50**	**Cries, covers ears or runs away upon hearing loud noises**	39 (37.50)	17 (48.57)	0.32	0.45 (0.36-0.053)
**51**	**Frustrating emotions**	64 (61.54)	22 (62.86)	1.00	0.49 (0.41-0.58)
**52**	**Hard to soothe when upset**	47 (45.19)	15 (42.86)	0.85	0.51 (0.42-0.60)

**Table AIII:** Univariate correlation values of variables and area under receiver operating curve (AuROC) and 95% confidence interval (CI) from clinical observations

**No.**	Clinical observations	ASD *N* (%)	Non-ASD *N* (%)	*p* value	AuROC (95% CI)
**1**	**The child’s eye contact is abnormal**	69 (66.35)	14 (40.00)	<0.01	0.63 (0.55-0.71)
**2**	**Gestures and words the child uses to approach you look weird**	78 (75.00)	16 (45.71)	<0.01	0.65 (0.56-0.73)
**3**	**When the child’s name is called (without touching), (s)he turns to look at you**	44 (42.31)	28 (80.00)	<0.01	0.69 (0.61-0.77)
**4**	**When you call to the child and signal with your eyes for him/her to look at things far away (without touching), (s)he looks in your eyes’ direction to those things**	43 (40.38)	30 (85.71)	<0.01	0.73 (0.65-0.80)
**5**	**If you hold a toy the child wants in your hand, (s)he speaks or make gestures along with eye contact to ask for it**	39 (37.50)	25 (71.43)	<0.01	0.67 (0.58-0.75)
**6**	**Requesting eye contact, speech and gestures are simultaneous (natural)**	27 (25.96)	19 (54.29)	<0.01	0.64 (0.56-0.72)
**7**	**Upon getting toys, the child shows off/shows it to you or guardian**	28 (26.92)	24 (68.57)	<0.01	0.71 (0.62-0.78)
**8**	**While playing with toys, the child tries to get attention so that you or guardian become(s) interested in what (s)he is interested in (for mutual interest)**	28 (26.92)	20 (57.14)	<0.01	0.65 (0.57-0.70)
**9**	**When you ask the child to play with toys (s)he likes, (s)he has fun with you**	48 (46.15)	24 (68.57)	0.03	0.61 (0.53-0.69)
**10**	**The child often interacts with you or guardian in examination room, such as makes eye contact, smiles at you or the guardian, initiates conversations or asks questions**	24 (23.08)	27 (77.14)	<0.01	0.77 (0.69-0.83)
**11**	**Overall, you can build a natural relationship with the child**	27 (25.96)	21 (60.00)	<0.01	0.67 (0.58-0.75)
**12**	**The child has language development for age (can say short phrases by two years of age, can say short sentences by three, can say several consecutive sentences by four)**	16 (15.38)	16 (45.71)	<0.01	0.63 (0.54-0.71)
**13**	**The child has natural speaking/tone of voice (that does not sound weird)**	22 (21.15)	16 (45.71)	<0.0.01	0.62 (0.54.0.71)
**14**	**The child shows emotions through facial expressions that look natural**	50 (48.08)	25 (71.43)	0.02	0.62 (0.53-0.70)
**15**	**The child uses gestures in communication (such as makes gestures in story-telling, shakes his/her head, nods or waives his/her hand in rejection)**	37 (35.58)	24 (68.57)	<0.01	0.67 (0.58-0.75)
**16**	**If you hold one toy in each hand and ask the child which toy (s)he wants, (s)he can point index finger to the toy (s)he wants on his/her own without having to be told**	38 (36.54)	26 (74.29)	<0.01	0.69 (0.61-0.77)
**17**	**The child often covers ears with hands**	7 (6.73)	0 (0.00)	0.19	0.53 (0.45-0.62)
**18**	**The child stares at lights, illuminating objects or rotating objects for a long time**	9 (8.65)	2 (5.71)	0.73	0.52 (0.43-0.60)
**19**	**The child smells or licks objects/people**	12 (11.54)	1 (2.86)	0.18	0.54 (0.46-0.63)
**20**	**The child makes certain repeated gestures (such as flick of the hand, tiptoeing, body rotation, moving fingers near face)**	20 (19.23)	3 (8.57)	0.19	0.55 (0.47-0.64)
**21**	**The child makes noises or strange speech (such as alien language, suddenly speaking out sounds from TV)**	65 (62.50)	10 (28.57)	<0.01	0.67 (0.58-0.75)
**22**	**The child repeats the sentence you just finished saying**	25 (24.04)	7 (20.00)	0.82	0.52 (0.43-0.60)
**23**	**The child does not know how to play with toys or to play with them as per their intended purposes (such as arranges, rotates or taps them repeatedly without role-play)**	33 (31.73)	7 (20.00)	0.20	0.56 (0.48-0.65)
**24**	**The child is interested in a particular part of objects (such as repeatedly spins car wheel without moving the car or is interested in repeatedly opening and closing doll’s eyes)**	26 (25.00)	6 (17.14)	0.49	0.57 (0.48-0.66)
**25**	**The child is more interested in certain objects in examination room than people**	71 (68.27)	12 (34.29)	<0.01	0.67(0.58-0.75)

## Appendix

Table AI

Table AII

Table AIII
